# Complete Genome Sequence of Ebrios, a Novel T7virus Isolated from the Ebrie Lagoon in Abidjan, Côte d’Ivoire

**DOI:** 10.1128/genomeA.00280-18

**Published:** 2018-06-21

**Authors:** Solange Ngazoa-Kakou, Cécile Philippe, Denise M. Tremblay, Stéphanie Loignon, Aristide Koudou, Ahmed Abole, David Ngolo Coulibaly, Stéphane Kan Kouassi, Mireille Kouamé Sina, Serge Aoussi, Mireille Dosso, Sylvain Moineau

**Affiliations:** a Plateforme de Biologie Moléculaire, Département Technique & Technologie, Institut Pasteur de Côte d’Ivoire, Abidjan, Côte d’Ivoire; b Département de biochimie, de microbiologie et de bio-informatique, Faculté des sciences et de génie, Québec, Canada; c Félix d’Hérelle Reference Center for Bacterial Viruses and GREB, Faculté de médecine dentaire, Université Laval, Québec, Canada

## Abstract

The lytic Escherichia coli phage Ebrios was isolated from a water sample collected in Ebrie Lagoon on the Adiopodoumé River in Abidjan (Republic of Côte d’Ivoire, West Africa). The linear genome of this Podoviridae family member contains 39,752 bp, has a G+C content of 52.9%, is composed of 53 open reading frames, and is related to the Stenotrophomonas maltophilia phage IME15.

## GENOME ANNOUNCEMENT

Lytic phages are currently being reconsidered as alternatives or complements to antibiotics (1) in many countries, including West Africa (2). Here, a new virulent phage was isolated from a water sample collected in April 2016 from Ebrie Lagoon (Abidjan, Republic of Côte d’Ivoire, West Africa). Briefly, the water sample was filtered (0.45 µm) and propagated in the Escherichia coli C strain using Luria Bertani (LB) broth and incubated overnight at 37°C (3). Several plaque morphologies were visible on LB plates, but, based on genomic DNA restriction profiles, only one distinct phage was isolated. A single plaque was purified three times, and the phage isolated was designated Ebrios. Twenty-seven bacterial strains were screened to establish the host range of phage Ebrios, consisting of Escherichia coli (4 strains), Salmonella (23 strains), and Stenotrophomonas (1 strain). Phage Ebrios did not form plaques on plates of any of the additional tested strains.

Phage Ebrios particles were stained with uranyl acetate (2%) and observed under an electron microscope (4). Electron micrographs revealed an icosahedral capsid (64 ± 2 nm in diameter) with a short tail, suggesting that phage Ebrios belongs to the Podoviridae family.

The genomic DNA of phage Ebrios was extracted from a high-titer lysate (10^9^ PFU · ml^−1^) using a plasmid maxikit (Qiagen) with modifications (5). The sequencing library was prepared with the Nextera XT DNA library preparation kit (Illumina), according to the manufacturer’s instructions, and sequenced using a MiSeq reagent kit v2 (500 cycles; Illumina) on a MiSeq instrument. *De novo* assembly was performed with the Ray assembler version 2.2.0 (6). The sequences were analyzed using Geneious software version 11.0.5. Gene annotation was performed using the DNA Master (http://phagesdb.org/DNAMaster/) workflow with Glimmer (7), GeneMark (8), and ORF Finder. Open reading frames (ORFs) were also manually validated if they contained at least 30 amino acids, had an initiation codon (AUG, UUG, or GUG), and were preceded by a Shine-Dalgarno sequence.

The genome of phage Ebrios has a G+C content of 52.9% and is composed of 39,752 bp and 53 open reading frames. The Ebrios genome is highly related to the genomes of Stenotrophomonas maltophilia phage IME15 (9) and E. coli podophage T7 (10), with average nucleotide identity (ANIb) values, calculated using JSpeciesWS (11), of 95.27% (79.80% query coverage) and 76.85% (66.36% query coverage), respectively. The first nucleotide of the Ebrios genome was defined based on its alignment with the phage IME15 genome.

Functions of gene products were assigned based on results from BLASTP searches (12). Twenty-nine proteins were annotated with a specific function. Bioinformatics analysis indicated that 44 of 53 deduced Ebrios proteins are related to proteins found in the S. maltophilia phage IME15. Four of the remaining proteins have homologs in other podophages, namely ORF9 (Yersinia phage phiYeO3-12), ORF18 (*Enterobacteria* phage 13a), ORF35 (E. coli phage CICC 80001), and ORF42 (E. coli phage EG1). ORF10 appears to be unique to phage Ebrios. Other predicted proteins, such as tail fiber (ORF42) or capsid (ORF35) proteins, were similar to those of T7viruses that infect E. coli.

Phage Ebrios was deposited in the Félix d’Hérelle Reference Center for Bacterial Viruses (www.phage.ulaval.ca) under the catalog number HER552.

### Accession number(s).

The complete genome sequence of phage Ebrios is available in GenBank under the accession number MG966531.
